# On Chinese Trachyphloeini with description of four new species (Coleoptera, Curculionidae, Entiminae)

**DOI:** 10.3897/zookeys.974.56059

**Published:** 2020-10-07

**Authors:** Li Ren, Roman Borovec, Runzhi Zhang

**Affiliations:** 1 Key Laboratory of Zoological Systematics and Evolution, Institute of Zoology, Chinese Academy of Sciences, No. 1 Beichen West Road, Chaoyang District, Beijing 100101, China; 2 Czech University of Life Sciences Prague, Faculty of Forestry and Wood Sciences, Department of Forest Protection and Entomology, Kamýcká 1176, CZ-165 21 Praha 6-Suchdol, Czech Republic; 3 University of Chinese Academy of Sciences, Beijing 100049, China

**Keywords:** New taxa, taxonomy, *
Rhinodontodes
*, *
Rhinodontus
*, *
Trachyphloeosoma
*, weevil

## Abstract

*Rhinodontodes alashanensis***sp. nov.**, *Trachyphloeosoma honza***sp. nov.**, *T. jirka***sp. nov.**, and *T. martin***sp. nov.** are described from China, illustrated and compared with similar species. The genus *Rhinodontodes* and the species *Rhinodontodes subsignatus* Voss, 1967 and *Rhinodontus mongolicus* Borovec, 2003 are recorded from China for the first time. Keys to all Chinese genera of Trachyphloeini, and to the Chinese species of *Rhinodontodes* and *Trachyphloeosoma*, are provided.

## Introduction

The Trachyphloeini Gistel, 1848 is a medium-sized tribe of entimines containing small wingless, terricolous species with body size 1.3–6.8 mm, having limited ability to migrate. They are mostly xerothermophilous, associated with steppe habitats, xeric grasslands, stony or sandy places, ranging to sandy semideserts (Borovec 2009). Only species of three genera from East Asia, *Pseudocneorhinus* Roelofs, 1873, *Trachyphilus* Faust, 1887 and *Trachyphloeosoma* Wollaston, 1869, are collected from forest litter (O´Brien 1984, Sawada et al. 1999, Morimoto 2015). The tribe is known primarily from the Palaearctic region, with only several genera known also from North America, South Africa, and Oriental region, but the tribal position of South African Trachyphloeini must be clarified. In the Palaearctic Region, the tribe is distributed from the Iberian Peninsula and north-western Africa up to Japan; the genera *Trachyphloeus* Germar, 1817 and *Romualdius* Borovec, 2009 occur in the whole of Europe and eastwards up to Kazakhstan, but the majority of genera have a much smaller area. At present, 13 genera with 363 species are known from the Palaearctic region (Alonso-Zarazaga et al. 2017).

Chinese Trachyphloeini have not been mentioned in literature very often due their cryptic way of life and difficulties in their collection. The majority of the species of the tribe, 67%, are known from the western Palaearctic region; 25% are known from Japan due Morimoto´s revision of the genus *Trachyphilus* (Morimoto 2015), and only 8% are known from China (Alonso-Zarazaga et al. 2017). However, the Himalo-Chinese region is home to a distinctive, peculiar, and specific terricolous fauna of Trachyphloeini, although there are not as many species as in the western subregion. Study of material deposited in the Institute of Zoology in Beijing, as well as newly collected material by several specialists focused on litter associated beetles, has increased our knowledge of the Chinese fauna. The first part of the results of our examination of this very interesting material was published in a previous article (Ren et al., 2019); the present paper adds additional information about other genera of Trachyphloeini.

## Materials and methods

The body length of specimens was measured in profile from the anterior margin of the eyes to the apex of the elytra, excluding the rostrum, as customary for curculionids. Rostral length was measured in dorsal view from the anterior margin of eyes to the anterior margin of the epistome and the rostral width was its maximum width. Pronotal and elytral length was measured along the mid-line length in dorsal view, width was the maximum width as measured in dorsal view. Entire abdomens were separated from the specimens and were macerated in 10% KOH for 7–10 days to remove soft tissues. They were then washed in distilled water. Internal abdominal segments were carefully separated from each other. Dissected female genitalia were embedded in Solakryl BMX, and dried male genitalia were glued to the same mounting card as the insect. The terminology of the rostrum and the terminalia follows Oberprieler et al. (2014).

Acronyms for depositories of the material are as follows:

**IZCAS**Institute of Zoology, Chinese Academy of Sciences;

**NMPC**Národní muzeum, Prague, Czech Republic;

**RBSC** Roman Borovec collection, Sloupno, Czech Republic;

**ZIN**Zoological Institute of the USSR Academy of Sciences, Saint Petersburg, Russia.

## Taxonomy

### Rhinodontodes

Taxon classificationAnimaliaColeopteraCurculionidae

Voss, 1967

0289303B-B20A-56A6-A635-DC5E93C8B6EE

Rhinodontodes Voss, 1967: 276 (original description); Alonso-Zarazaga and Lyal 1999: 183 (catalogue); Borovec 2003: 31 (note); Borovec 2009: 76 (redescription of the genus); Alonso-Zarazaga et al. 2017: 403 (catalogue).

#### Remarks.

The genus was described by Voss (1967) for a single species, *Rhinodontodes subsignatus* Voss, 1967, based on a single specimen from Mongolia. The holotype was later examined by Borovec (2003) and until recently was the only specimen of the genus known. We now have access to 21 more specimens of this genus, mainly from the collections of IZCAS and ZIN, and we are able to discuss characters used for the definition of *Rhinodontodes*. Voss described the genus as similar to *Rhinodontus* in its elongated epistome, but distinguishable by tarsomere 3 being wider than tarsomere 2, claws parallel in basal half, apical part of protibiae not distinctly enlarged laterally, rounded, without spines and narrower pronotum, 1.34–1.42 × as wide as long, with anterior margin not distinctly narrower than posterior one. Borovec (2009) in his phylogenetic analysis of the tribe Trachyphloeini confirmed *Rhinodontodes* as related to *Rhinodontus* and *Pseudocneorhinus*, sharing the character states of epistome projected anteriorly and ocular lobes with short setae with *Rhinodontus*, and having as an autapomorphy, rostrum continuous with head, not separated by any furrow. Some of the characters previously used to distinguish *Rhinodontodes* are not unique, in comparison with newly known *Pseudocneorhinus* described in Ren et al. (2019). Males of *P. bifasciatus* Roelofs, 1880 also have the epistome projected anteriorly (Borovec 2009: figs 55, 61), creating a striking tooth, and *P. glaber* Ren, Borovec, Zhang, 2019 also have weak ocular lobes with very short setae and, especially, have a long rostrum and epifrons constricted in the middle. These two species thus show characters very similar to the shape of the rostrum of *Rhinodontodes*. Thus, *Rhinodontodes* seems to be more closely related to *Pseudocneorhinus* than was previously assumed. Study of further material of both genera will confirm whether the two genera are separate or should be placed in synonymy. Presently, *Rhinodontodes* can be distinguished from *Pseudocneorhinus* mainly by the rostrum and head being on the same level and the protibiae being laterally weakly enlarged (Borovec 2009: fig. 58).

### Rhinodontodes alashanensis sp. nov.

Taxon classificationAnimaliaColeopteraCurculionidae

120B163E-A81A-5166-9625-58D92A584CFE

http://zoobank.org/CEF06CD7-8A76-411D-BF30-4371F691F530

Figs 1–4 , 25 , 26–27 , 52

#### Type locality.

Alashan, Bayanhaotezhen (China: Inner Mongolia).

#### Material examined.

***Holotype.*** China – **Inner Mongolia Autonomous Region** • 1 ♂; Алашанъ, Дын-юань-ин, V.08 ек. Козлова [Alashan, Ding-yuan-ying (now Bayanhaotezhen), v.1908, Kozlov’s expedition]; Pseudocneorhinus alashanicus Typ. m.; G. Suvorov det.; ZIN. *Paratypes*. CHINA – **Inner Mongolia Autonomous Region** • 1 ♂ 1 ♀; same data as for holotype; ZIN.

#### Description.

Body length: 3.94–4.31 mm, holotype 3.94 mm.

**Figures 1–8.**
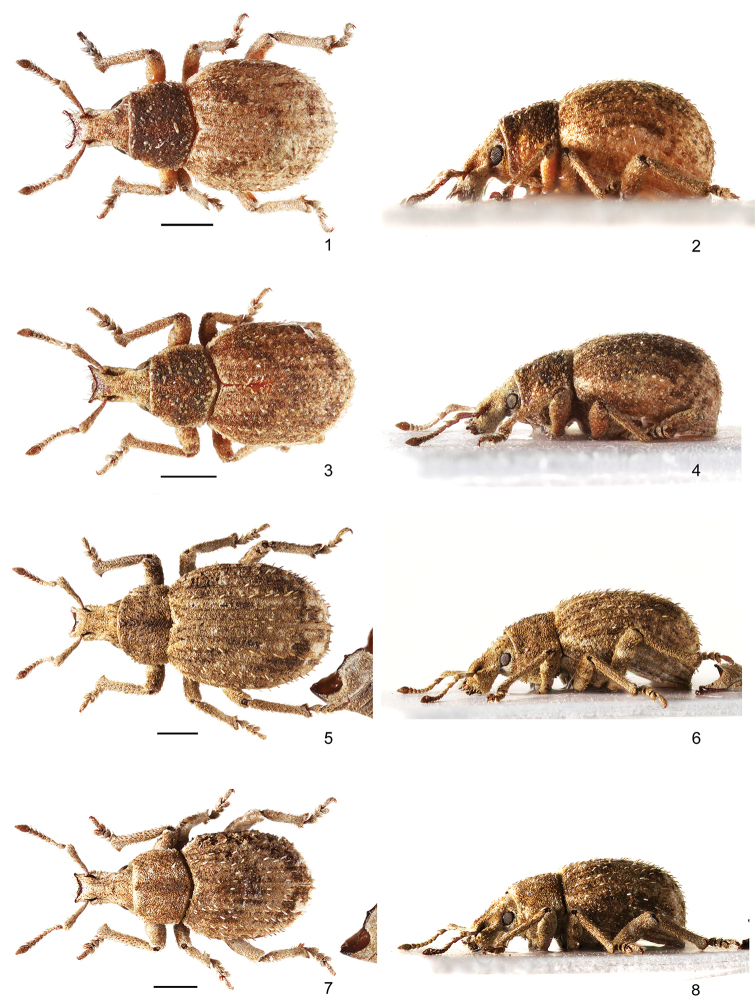
Habitus of species of *Rhinodontodes*: **1, 2***R. alashanensis* sp. nov., female, paratype, dorsal and lateral view **3, 4***R. alashanensis* sp. nov., male, holotype, dorsal and lateral view **5, 6***R. subsignatus*, female, dorsal and lateral view **7, 8***R. subsignatus*, male, dorsal and lateral view. Scale bars: 1 mm.

***Body*** (Figs 1–4) dark brownish, epistome, mucros, and claws reddish brown, fringe of setae on protibiae yellowish. Appressed scales covering antennae, head, pronotum, elytra and legs, except antennal clubs; scales on elytra oval, wider than long, densely and finely longitudinally striate, very dense, imbricate, six or seven scales across interval width, light brownish on disc with small, irregularly scattered dark brownish and greyish spots and with light greyish stripe along lateral margins, occupying three lateral intervals and very short apical part of elytra. Pronotum and head with rostrum with oval appressed scales standing on their edges and visible only as special structure of narrow short lines, only short flat area behind frons with the same appressed scales as elytra. Semi-appressed elytral setae subspatulate to spatulate, in holotype more slender than in paratypes, approximately as long as half of width of one interval, densely and finely longitudinally striate, creating one regular row on each interval, distance between two setae 2 × length of one seta. Pronotum and head with rostrum with almost identical semi-appressed setae, irregularly scattered, on pronotum transversely directed, on rostrum shorter than on elytra and longitudinally directed. Antennal scapes and femora with semi-appressed setae, funicles, tibiae and tarsi with semi-erect, moderately long setae, prominent from outline. Clubs densely and finely setose.

***Rostrum*** (Figs 1–4, 25) 1.17–1.22 × as long as wide, from base slightly, regularly enlarged apicad with straight sides, at apex only slightly wider than at base. Epifrons at basal third distinctly tapered apicad, then weakly enlarged apicad, in both parts with slightly convex sides, at apex distinctly narrower than at base, longitudinally shallowly depressed. Epistome long and conspicuous, distinct in dorsal and lateral view, as wide as apex of rostrum or slightly wider, separated from frons by very narrow carina, in females U-shaped, slender, lengthily exceeding anterior rostral margin, with tips directed anteriad, in males V-shaped, wider, less exceeding anterior rostral margin, with tips directed obliquely, laterally. Frons flat, squamose, bearing in lateral parts four or five pairs of stout apical setae, obliquely directed anteriad. Scrobes in dorsal view visible in apical third of rostrum as narrow furrows; in lateral view narrow, subparallel-sided, weakly curved, directed towards middle of eyes, visible as short furrow only in apical half of rostrum, in basal half with margins weakly indicated. Rostrum in lateral view somewhat convex, separated from head by shallow transverse depression. Eyes almost flat, hardly prominent from outline of head. Head distinctly enlarged basad.

***Antennae*** slender; scapes faintly regularly curved, approximately equally long as funicles, at basal two thirds weakly and regularly enlarged apicad, at apical third enlarged somewhat more, at apex equally wide as clubs. Funicles with segments 1 and 2 conical, long, funicle segment 1 slightly longer and wider than segment 2, in males more slender than in females; in males funicle 1 1.7–1.8 × as long as wide; segment 2 1.8–1.9 × as long as wide; segment 3 1.1 × as long as wide; segments 4 and 5 isodiametric; segment 6 1.1 × as wide as long; segment 7 1.4 × as wide as long; in females funicle 1 1.7–1.8 × as long as wide; segment 2 1.6–1.7 × as long as wide; segment 3 and 4 1.2 × as wide as long; segment 5 1.3 × as wide as long; segment 6 1.4 × as wide as long; segment 7 1.6 × as wide as long.

***Pronotum*** (Figs 1–4) 1.34–1.36 × as wide as long, widest just behind the midlength, with rounded sides, more strongly tapered anteriad than posteriad, behind anterior margin weakly constricted. Disc regularly convex. Base arched. Pronotum in lateral view moderately convex, ocular lobes developed.

***Elytra*** (Figs 1–4) 1.26–1.30 × as long as wide, oval, widest at midlength, with regularly rounded sides; shoulders regularly rounded; basal margin arched. Striae narrow, punctured, punctures hidden by appressed scales; stria 1 at base distinctly curved outwards, sutural interval at base distinctly enlarged. Interval almost flat, equally wide and elevated. Elytra in lateral view convex.

***Protibiae*** moderately long and slender, mesally distinctly, laterally weakly enlarged, at apex rounded, with fringe of short and fine yellowish setae, mucronate, inner margin of protibiae and metatibiae with 2–3 very small, black, almost indistinct teeth; metatibial corbels densely squamose. Tarsi slender; tarsomere 2 1.2–1.3 × as wide as long; tarsomere 3 1.5–1.6 × as wide as long and 1.4 × as wide as tarsomere 2; onychium (tarsomere 5) 1.4–1.6 × as long as tarsomere 3. Claws fused at basal third, moderately and regularly divergent apicad.

***Penis*** (Fig. 26) short and wide, 1.91 × as long as wide, in ventral view at base and at apex approximately equally wide, parallel-sided with slightly concave sides; apex regularly rounded to small, regular triangular prolongation; in lateral view wide, regularly curved, subcrescent-shaped, with slender and short apical elongation.

***Female genitalia.*** Sternite VIII umbrella-shaped with short apodeme. Gonocoxites not examined. Spermatheca (Fig. 28) with long, regularly and distinctly curved cornu; corpus large; ramus subsquare, nodulus smaller, subtriangular.

#### Biology.

Unknown.

#### Distribution.

China, Inner Mongolia (Fig. 52).

#### Etymology.

Patronymic, name is derived from the name of type locality.

#### Differential diagnosis.

*Rhinodontodes alashanensis* is similar to the only other known species of the genus, *R. subsignatus* Voss, 1967. It is possible to distinguish the two species by the following key:

**Table:** 

1	Larger body size, 4.5–5.4 mm. Epistome short, with points narrower than rostrum at apex, in females V-shaped, moderately robust, slightly exceeding anterior margin of rostrum (Figs 5–8). Pronotum wider, 1.37–1.41 × as wide as long (Figs 5–8). Penis with long apical part (Fig. 27). China, Inner, Mongolia; Mongolia	***R. subsignatus* Voss**
–	Smaller body size, 3.9–4.3 mm. Epistome long, with tips as wide or wider than rostrum at apex, in females U-shaped, slender, distinctly exceeding anterior margin of rostrum (Figs 1–4). Pronotum narrower, 1.34–1.36 × as wide as long (Figs 1–4). Penis with short apical part (Fig. 26). China, Inner Mongolia	***R. alashanensis* sp. nov.**

### Rhinodontodes subsignatus

Taxon classificationAnimaliaColeopteraCurculionidae

Voss, 1967

32750428-A5F5-5BD7-A688-5125582A097B

Figs 5–8 , 27 , 29 , 52

Rhinodontodes subsignatus Voss, 1967: 277 (original description); Alonso-Zarazaga and Lyal 1999: 183 (catalogue); Borovec 2003: 49 (type examination); Borovec 2009: 76 (check-list); Alonso-Zarazaga et al. 2017: 403 (catalogue).

#### Material examined.

China – **Inner Mongolia Autonomous Region** • 2 ♂♂ 2 ♀♀; 阿拉善左旗贺兰山水磨沟正沟 [Alxa Zuoqi, Helan Mountains, Shuimogou Zhenggou]; 27 Jul. 2010; 黄鑫磊 [X.L. Huang leg.]; IZCAS, IOZ(E)1965104, IOZ(E)1965108, IOZ(E)1965105, IOZ(E)1965110; • 3 ♀♀; 阿拉善左旗贺兰山哈拉乌青树湾 [Alxa Zuoqi, Helan Mountains, Halawu, Qingshuwan]; 30 Jul. 2010; 黄鑫磊 [X.L. Huang leg.]; IZCAS, IOZ(E)1965107, IOZ(E)1965109, IOZ(E)1965113; • 1 ♀; 阿拉善左旗贺兰山主峰峰顶 [Alxa Zuoqi, the main top of Helan Mountains]; 3134 m a.s.l.; 17 Aug. 2010; 38°49.8'N, 105°56.4'E; 林美英 [M.Y. Lin leg.]; IZCAS, IOZ(E)1965112; • 1 ♂; 阿拉善左旗贺兰山水磨沟正沟 [Alxa Zuoqi, Helan Mountains, Shuimogou Zhenggou]; 2025 m a.s.l.; 16 Aug. 2010; 38°55.8'N, 105°53.4'E; 林美英 [M.Y. Lin leg.]; IZCAS, IOZ(E)1965114 • 2 ♀♀; 阿拉善左旗贺兰山强岗岭 [Alxa Zuoqi, Helan Mountains, Qianggangling]; 8 Aug. 2010; 黄鑫磊 [X.L. Huang leg.]; IZCAS, IOZ(E)1965106, IOZ(E)1965111; • 1 ♂ 1 ♀; 阿拉善左旗贺兰山古拉本 [Alxa Zuoqi, Helan Mountains, Gulaben]; 6 Aug. 2010; 黄鑫磊 [X.L. Huang leg.]; IZCAS, IOZ(E)1965102, IOZ(E)1965101; • 1 ♀; 阿拉善左旗贺兰山北寺 [Alxa Zuoqi, Helan Mountains, Beisi]; 13 Aug. 2010; 黄鑫磊 [X.L. Huang leg.]; IZCAS, IOZ(E)1965103; • 1 ♀; 阿拉善左旗贺兰山水磨沟 [Alxa Zuoqi, Helan Mountains, Shuimogou]; 25 Jul. 2010; 黄鑫磊 [X.L. Huang leg.]; IZCAS, IOZ(E)1941122.

Mongolia • 1 ♀; 40 km W Dalanzadgad, Gobi Gurvansaikhan NP, Yolyn am env.; 28–30 Jun. 2003; 1700–2000 m a.s.l.; Z. Jindra leg.; RBSC; • 2 ♀♀; Bayan-Chong, Aimak, Ich-Bogdo-Ula, srednegorie [central mountains]; 2500 m a.s.l.; 3 Jul. 1973; G. Medvedev leg.; ZIN.

#### Remarks.

The eighteen specimens examined of *R. subsignatus* come from Mongolia and also from China, Inner Mongolia Autonomous Region. The four males and 11 females from China differ somewhat from the three females from Mongolia, which share with the holotype slender, subparallel-sided, semi-erect elytral setae, while material from Mongolia has wider, subspatulate, semi-appressed elytral setae. Mongolian and Chinese specimens are almost identical in all other characters thus we assume the shape of elytral setae is a variable character of the species. This is the first record of *R. subsignatus* from China (Fig. 52).

### Rhinodontus

Taxon classificationAnimaliaColeopteraCurculionidae

Faust, 1890

4F2E798D-084C-52C8-AB1A-D9D36F22590F

Rhinodontus Faust, 1890: 455 (original description); Alonso-Zarazaga and Lyal 1999: 183 (catalogue); Borovec 2003: 32 (genus revision); Borovec 2009: 76 (check-list); Alonso-Zarazaga et al. 2017: 403 (catalogue).

#### Remarks.

The genus is well defined and distinguished by apex of protibiae strikingly enlarged laterally, armed with wide spines, epistome long, rostrum short and wide, distinctly enlarged before eyes and body wide and robust. It was described as monotypic by Faust (1890) and studied later by Voss (1967) and Borovec (2003). *Rhinodontus* currently contains five valid species from China and Mongolia (Alonso-Zarazaga et al. 2017). Among material from IZCAS and ZIN we discovered specimens which add to our knowledge distribution of the species, which were previously known from only a limited number of specimens.

### Rhinodontus crassiscapus

Taxon classificationAnimaliaColeopteraCurculionidae

Borovec, 2003

BBA534C3-2FA6-51F7-882F-7C4DA8294097

Figs 9 , 10 , 30

Rhinodontus crassiscapus Borovec, 2003: 38 (original description); Borovec 2009: 76 (check-list); Alonso-Zarazaga et al. 2017: 403 (catalogue).

#### Material examined.

China – **Qinghai Prov.** • 2 ♀♀; Ю. скл. хр. Бурхан-Будда: дoл. oз. Алык-нoр. 30.V.1900. Експ. Кoзлoва. [southern slope of the mountains Burchan-Buddha, valley of the lake Alake Hu. 30.v.1900; Kozlov’s expedition]; ZIN.

#### Remarks.

This species was described based on three females from China, Xinjiang and Gansu. This is the first additional locality since the original description.

*Rhinodontus crassiscapus* differs from all other species of the genus by its very short, distally thickened scape and by its long raised elytral setae being longer than one half of the interval width.

**Figures 9–16.**
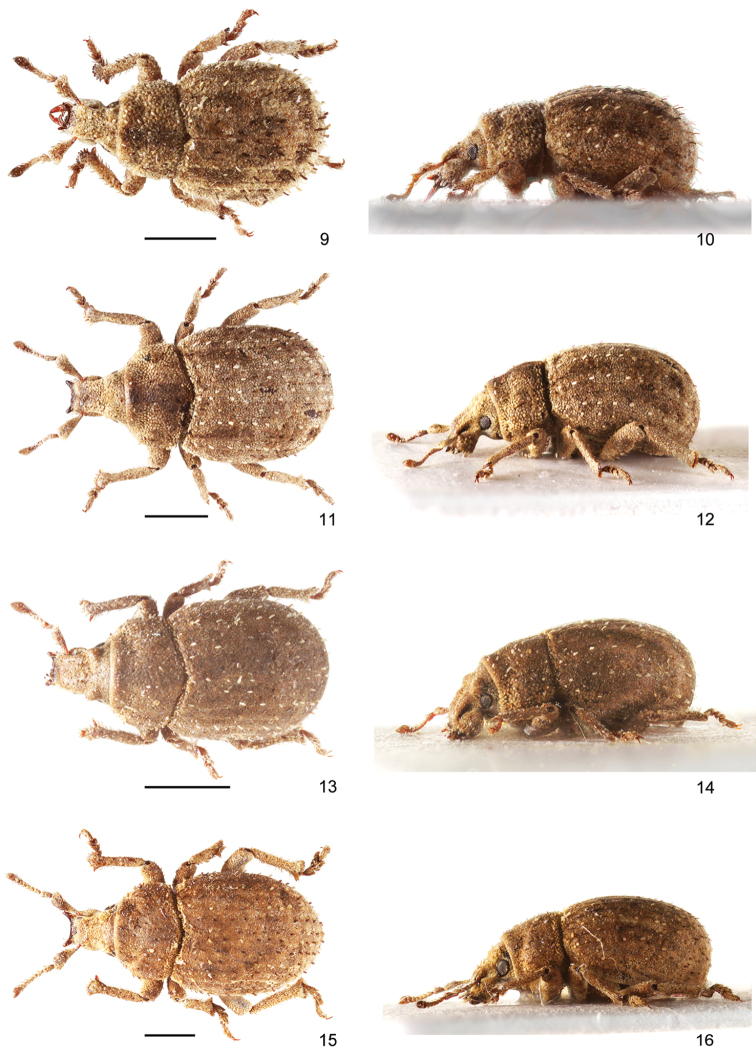
Habitus of species of *Rhinodontus*: **9, 10***R. crassiscapus*, female, dorsal and lateral view **11, 12***R. ignarus*, female, dorsal and lateral view **13, 14***R. mongolicus*, female, dorsal and lateral view **15, 16***R. proximus*, female, dorsal and lateral view. Scale bars: 1 mm.

### Rhinodontus ignarus

Taxon classificationAnimaliaColeopteraCurculionidae

Faust, 1890

8F6F4056-F0C6-5E77-BDE5-EF41EBA5E089

Figs 11 , 12 , 31 , 32

Rhinodontus ignarus Faust, 1890: 455 (original description); Alonso-Zarazaga and Lyal 1999: 183 (catalogue); Borovec 2003: 32 (redescription); Borovec 2009: 76 (check-list); Alonso-Zarazaga et al. 2017: 403 (catalogue).
Rhinodontus proximus centralis Voss, 1967: 276 (original description).

#### Material examined.

China – **Inner Mongolia Autonomous Region** • 2 ♀♀; 阿拉善左旗贺兰山哈拉乌青树湾 [Alxa Zuoqi, Helan Mountains, Halawu, Qingshuwan]; 30 Jul. 2010; 黄鑫磊 [X.L. Huang leg.]; IZCAS, IOZ(E)1965140, IOZ(E)1965142; • 1 ♀; 阿拉善左旗贺兰山强岗岭 [Alxa Zuoqi, Helan Mountains, Qianggangling]; 8 Aug. 2010; 黄鑫磊 [X.L. Huang leg.]; IZCAS, IOZ(E)1965141; • 6 ♀♀; 阿拉善左旗贺兰山哈拉乌主峰 [Alxa Zuoqi, Helan Mountains, the main top of Halawu]; 1 Aug. 2010; 黄鑫磊 [X.L. Huang leg.]; IZCAS, IOZ(E)1965143–1965148.

Mongolia • 1 ♀, Centralnyi Aimak, Dzorgol-Khairkhan, 30 km NE Undzhul; 16 Jul. 1973; G. Medvedev leg.; ZIN; • 1 ♀; Centralnyi Aimak, Dzorgol-Khairkhan, Uver-Undzhul-Ul hill; 16 Jul. 1973; G. Medvedev leg.; ZIN.

#### Remarks.

Nine females from China (Inner Mongolia) have the spermatheca with a shorter ramus and more slender collum and nine spines at the protibial apex in comparison with previously known material, including the type specimens, of the species having only eight spines. Due to the lack of males of this population we currently retain it as conspecific with *R. ignarus*.

### Rhinodontus mongolicus

Taxon classificationAnimaliaColeopteraCurculionidae

Borovec, 2003

47E16381-005D-55D2-90D3-30D68AFE87AA

Figs 13 , 14 , 33 , 53

Rhinodontus mongolicus Borovec, 2003: 36 (original description); Borovec 2009: 76 (check-list); Alonso-Zarazaga et al. 2017: 403 (catalogue).

#### Material examined.

China – **Inner Mongolia Autonomous Region** • 1 ♀; 阿拉善左旗贺兰山哈拉乌主峰[Alxa Zuoqi, Helan Mountains, the main top of Halawu]; 1 Aug. 2010; 黄鑫磊 [X.L. Huang leg.]; IZCAS, IOZ(E)1965118; • 1 ♀; 阿拉善左旗贺兰山强岗岭 [Alxa Zuoqi, Helan Mountains, Qianggangling]; 8 Aug. 2010; 黄鑫磊 [X.L. Huang leg.]; IZCAS, IOZ(E)1965131; • 1 ♀; 阿拉善左旗贺兰山水磨沟正沟 [Alxa Zuoqi, Helan Mountains, Shuimogou Zhenggou]; 27 Jul. 2010; 黄鑫磊 [X.L. Huang leg.]; IZCAS, IOZ(E)1965133; • 1♀; 阿拉善左旗贺兰山哈拉乌青树湾 [Alxa Zuoqi, Helan Mountains, Halawu, Qingshuwan]; 30 Jul. 2010; 黄鑫磊 [X.L. Huang leg.]; IZCAS, IOZ(E)1965137; • 1 ♀; 阿拉善左旗贺兰山水磨沟 [Alxa Zuoqi, Helan Mountains, Shuimogou]; 25 Jul. 2010; 黄鑫磊 [X.L. Huang leg.]; IZCAS, IOZ(E)1941074.

Mongolia • 1 ♀; Uver Khangaiskyi Aimak, Arc-Bogdo Mts., 20 km S Khovda; 12–13 Aug. 1967; Kerzhner leg.; ZIN; • 1 ♀; Ara-Khangaiskyi Aimak, 20 km NE Tevshrulakh; 17 Jun. 1975; Emelianov leg.; ZIN; • 1 ♀; Uver Khangaiskyi Aimak, Orkhon, 15 km W Bat-Ulgyi; 22 Sept. 1981; Korolas leg.; ZIN.

#### Remarks.

The species was described based on 17 females from Mongolia, Ulaan Baatar and these are the first additional specimens since the original description. This is also the first record of the species in China (Fig. 53). *Rhinodontus mongolicus* is very easy distinguishable from all other species of *Rhinodontus* by the prominent sulci covering eyes in dorsal view and by the slender antennal scape.

### Rhinodontus proximus

Taxon classificationAnimaliaColeopteraCurculionidae

Voss, 1967

5248D370-C7EE-5AEC-83FF-00CE501EE92D

Figs 15 , 16 , 34

Rhinodontus proximus Voss, 1967: 275 (original description); Borovec 2003: 34 (redescription); Borovec 2009: 76 (check-list); Alonso-Zarazaga et al. 2017: 403 (catalogue).

#### Material examined.

China – **Inner Mongolia Autonomous Region** • 1 ♀; 阿拉善左旗贺兰山南寺雪岭子[Alxa Zuoqi, Helan Mountains, Nansi, Xuelingzi]; 11 Aug. 2010; 黄鑫磊 [X.L. Huang leg.]; IZCAS, IOZ(E)1965149; • 4 ♀♀; 阿拉善左旗水磨沟 [Alxa Zuoqi, Helan Mountains, Shuimogou]; 25 Jul. 2010; 黄鑫磊 [X.L. Huang leg.]; IZCAS, IOZ(E)1941092–1941095. – **Gansu Prov. [Kan-Ssu Prov.**] • 1 ♀; 1884; O. Potanin leg.; ZIN.

Mongolia • 1 ♀; Bayankhongor aym., Khangayan Nuruu Mts., Tsagaan-Ovoo 25 km W; 45°55.1'N, 101°10.4'E; 2050 m, a.s.l.; 8 Jun. 2013; M. Košťál leg.; MKBC; • 2 ♀♀; Uver Khangaisk Aimak, Arc-Bogdo Mts., 20 km S Khovda; 12–13 Aug. 1967; Kerzchner leg.; ZIN; • 2 ♀♀; Iuzhno-Gob. Aimak, Ukh-Shankhai; 12 Jun. 1972; ZIN; • 3 ♀♀; Iuzhno-Gob. Aimak, 25 km SW Bulgan; 5 Aug. 1971; ZIN; • 1 ♀; Iuzhno-Gob. Aimak, Navtgar-Ul hill, 35 km NW Iamat-Ul; 9 Aug. 1971; Emelianov leg.; ZIN; • 3 ♀♀; Vostochno-Gob. Aimak, Nomt-Ul hill, 30 km SSE Shokhoi-Nur lake; 26 Jun. 1971; Emelianov & Kozlov leg.; ZIN; • 1 ♀; Baian-Kchongor. Aimak, 20 km ESE Uldzint; 9 Jul. 1970; Emelianov leg.; ZIN; • 2 ♀♀; Iuzhno-Gob. Aimak, Tachilga-Ul hill, 35 km NNE Dalan-Deadagad; 10 Aug. 1971; Kerzhner leg.; ZIN; • 1 ♀; Centralnyi Aimak, Dzorgol-Khairkhan, Uver-Undzhul-Ul hill; 16 Jul. 1973; G. Medvedev leg.; ZIN; • 1 ♀; Iuzhno-Gob. Aimak, Khuryn-Khalkha-Nur, 25 km W Noën; 20 Jun. 1973; G. Medvedev leg.; ZIN.

#### Remarks.

This species was described from four specimens from two localities in Mongolia, later recorded also from China. It is very similar to *R. ignarus*, but differs by possessing eight or nine spines at apex of protibia, tarsal claws connate only in the very short basal part, and also the more slender antenna.

### Rhinodontus sawadai

Taxon classificationAnimaliaColeopteraCurculionidae

Borovec, 2003

69834EBD-F4CA-58B8-89DE-37081AC6336C

Figs 17 , 18 , 35

Rhinodontus sawadai Borovec, 2003: 40 (original description).
Rhinodontus sawadai : Borovec 2009: 76 (check-list); Alonso-Zarazaga et al. 2017: 403 (catalogue).

#### Material examined.

China – **Xinjiang Autonomous Region** • 1 ♀; Polu; 13 May 1890; ZIN.

#### Remarks.

This species was described based on three females from China, Xinjiang; this is the first additional specimen since the original description. *Rhinodontus sawadai* can be distinguished from other species of the genus by its wider rostrum, curved scape, missing prominences above eyes, and less enlarged outside apex of protibia.

**Figures 17–24.**
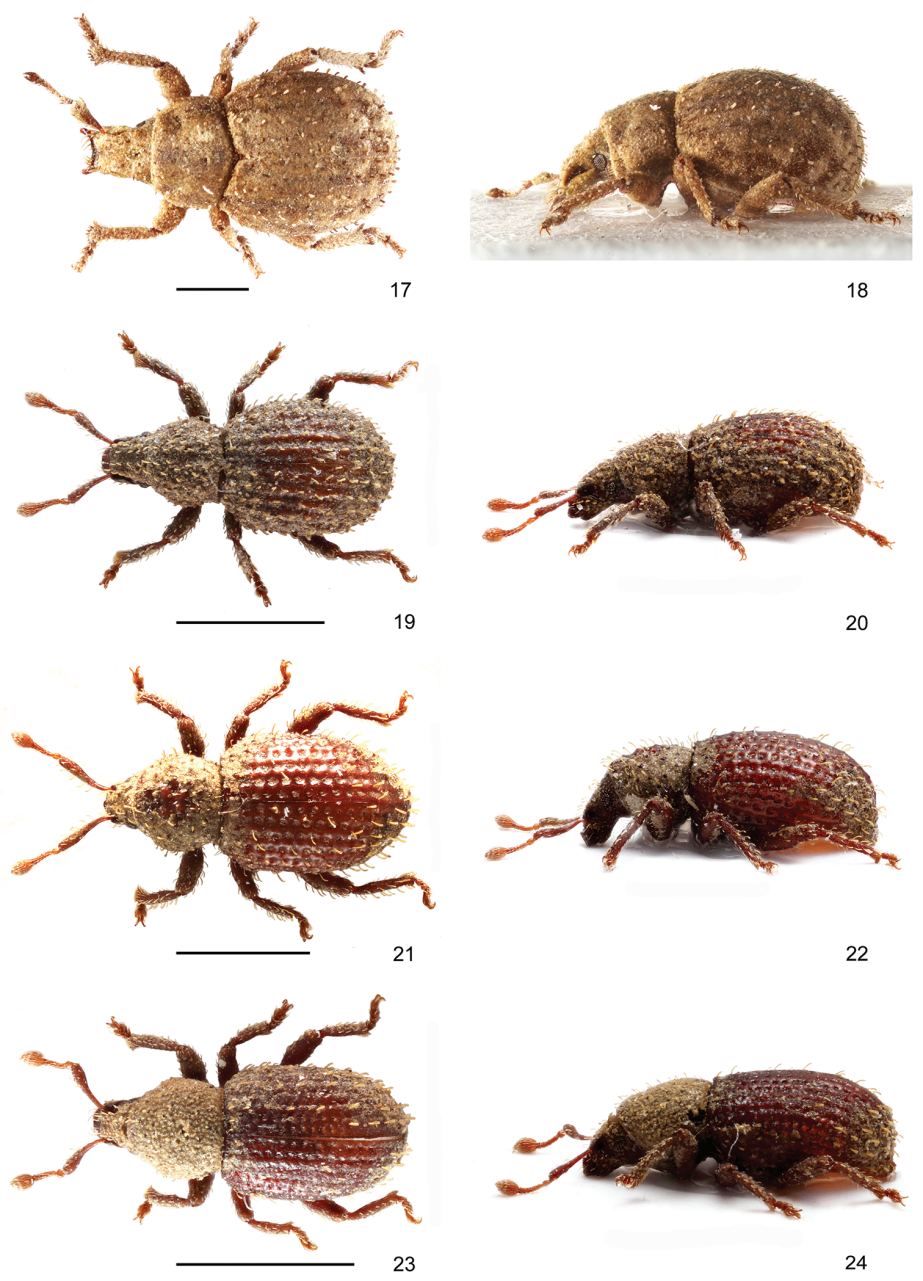
Habitus of species of *Rhinodontus* and *Trachyphloeosoma*: **17, 18***Rhinodontus sawadai*, female, dorsal and lateral view **19, 20***Trachyphloeosoma honza* sp. nov., paratype, female, dorsal and lateral view **21, 22***T. jirka* sp. nov., paratype, female, dorsal and lateral view **23, 24***T. martin* sp. nov., paratype, female, dorsal and lateral view. Scale bars: 1 mm.

### Trachyphloeosoma

Taxon classificationAnimaliaColeopteraCurculionidae

Wollaston, 1869

2D61DFD1-3A97-57E0-AD58-904BEFFD9632

Trachyphloeosoma Wollaston, 1869: 414 (original description); Zimmerman 1956: 27 (review of genus); Alonso-Zarazaga and Lyal 1999: 183 (catalogue); Borovec 2009: 52 (redescription of genus); Borovec 2014: 11 (revision of genus); Morimoto 2015: 343 (review of Japanese species); Alonso-Zarazaga et al. 2017: 406 (catalogue).

#### Remarks.

This genus was described by Wollaston based on material from the island of St. Helena. Additional species were described later, and the present number of valid species is five. The genus was redescribed and compared to all other Palaearctic Trachyphloeini by Borovec (2009), then subsequently revised by Borovec (2014), based on material from China, Vietnam, Japan, Korea, and the Moluccas. Morimoto (2015), in his monography of Japanese Entiminae, surveyed the Japanese species of the genus. China is the most northwestern part of the range of the genus, and *Trachyphloeosoma* was first recorded from this country only in 2009 by Borovec, based on one male from Yunnan, and subsequently by Borovec (2014) based on several additional specimens of the same species. Following examination of some newly sifted material from China, and comparing this with Morimoto’s (2015) review of the genus from the Japanese islands, we can state that the species collected in China are new to science. We can thus correct the name of the species so far recorded from China. This newly collected material from China was erroneously identified as *T. advena* Zimmerman, 1956 and was listed under this name in the Palaearctic catalogue (Alonso-Zarazaga et al. 2017). After dissection and thorough examination, we are able to recognise the specimens as distinct from *T. advena* and belonging to three different species, which are described below.

**Figure 25.**
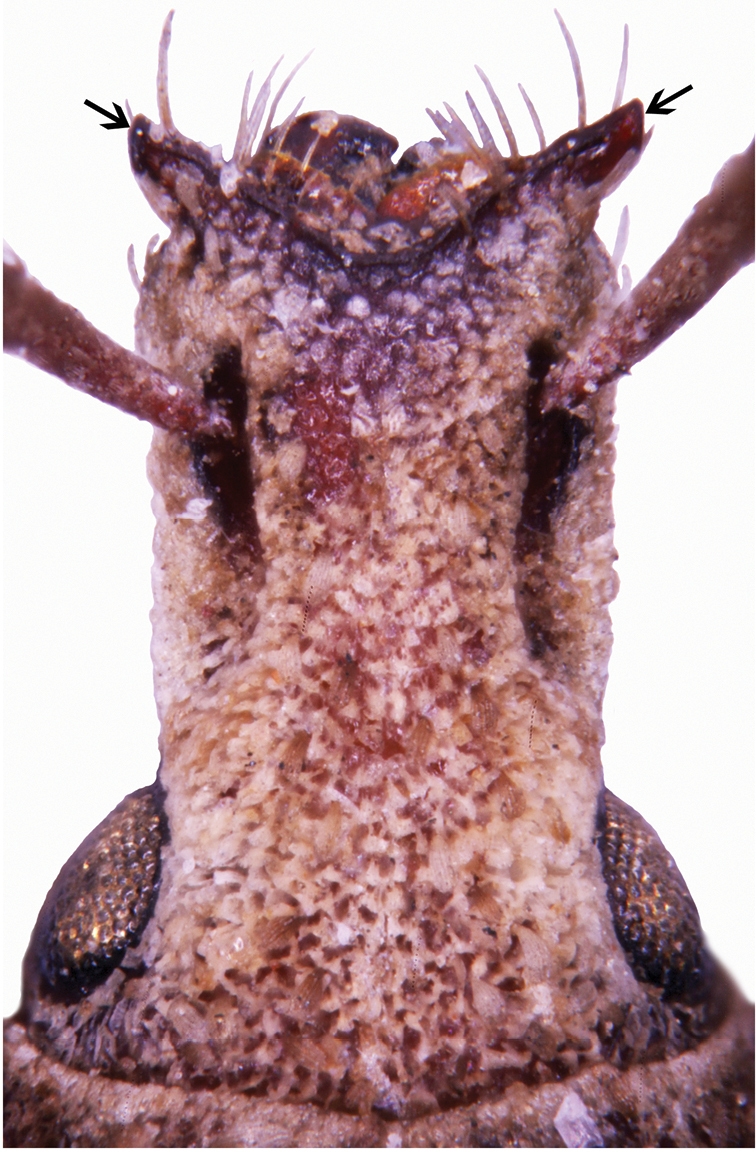
Head of *Rhinodontodes alashanensis*, sp. nov., dorsal view, arrows indicate the epistome.

### Trachyphloeosoma honza sp. nov.

Taxon classificationAnimaliaColeopteraCurculionidae

BB670A72-941D-5BB0-A8DD-BECF56913C3A

http://zoobank.org/F296C5F2-B37B-4170-A85F-581BC127CD91

Figs 19 , 20 , 36 , 40 , 42 , 44 , 48 , 54

Trachyphloeosoma advena : Borovec 2009: 78 (check-list); Borovec 2014: 12 (note); Alonso-Zarazaga et al. 2017: 406 (catalogue). **non** Zimmermann, 1956.

#### Type locality.

China, Yunnan, Lunan.

#### Material examined.

***Holotype.*** China – **Yunnan Prov.** • 1 ♂; Lunan – env., Stone Forest; 29 Jul. 1995; Z. Jindra leg.; NMPC. *Paratypes*. CHINA – **Yunnan Prov.** • 10 ♀♀; 14 km SE Tengchong, Renjiafen env.; 24°56.43'N, 98°35.52'E; 2145 m a.s.l.; (CH06) 23 Jun. 2016; J. Hájek & J. Růžička leg.; sift ♯05, border of old orchard, wet debris under trees; NMPC; • 2 ♀♀; same data as for preceding; RBSC; • 1 ♀; same data as for preceding; IZCAS; • 2 ♀♀; Tengchong city, Laifeng Shan Forest Park; 25°01.24'N, 98°28.94'E; 1800 m a.s.l.; (CH05) 22 Jun. 2016; J. Hájek & J. Růžička leg.; sift ♯04, dense mixed forest above tombs near track, wet debris in terrain depressions; NMPC.

#### Description.

Body length: 1.87–2.39 mm, holotype 2.13 mm.

***Body*** (Figs 19, 20) unicoloured piceous brown, antennae and legs slightly paler, reddish brown. The entire body except for frons, antennal funicles with clubs and tarsi covered with a brownish earth-like incrustation which conceals most of the surface; rounded appressed scales, covering the whole body, very hardly visible through this incrustation. Elytra with one conspicuous, dense row of erect, subspatulate setae on each interval, starting just from the base; setae approximately as long as half width of one interval, slightly enlarged apicad, distance between two setae slightly longer than length of one seta. Pronotum and head with rostrum with similar setae, less than half as long as elytral ones, densely irregularly scattered, anteriorly directed. Antennal scapes, femora and tibiae with long, erect, very slender setae, distinctly prominent from outline of scapes and legs.

***Rostrum*** (Figs 19, 20, 36) 1.25–1.31 × wider than long, at base 1.18–1.23 × wider than at apex, evenly tapered anteriad with almost straight sides, at short basal part with shallowly concave sides; in profile short and wide, convex. Epifrons distinctly tapered anteriad with straight sides, at level of antennal insertion narrow, 0.65–0.68 × as wide as corresponding width of rostrum, with ill-defined, shallow, longitudinal furrow. Frons conspicuous, glabrous, smooth and shiny, posteriorly continuous with epifrons. Epistome indistinct. Antennal scrobes in dorsal view fully visible as furrows, reaching eyes; in lateral view with dorsal margin directed towards middle of eye and ventral margin deeply below ventral margin of eye. Eyes small, in dorsal view protruding from outline of head; in lateral view placed in dorsal third, distance from dorsal margin of head longer than diameter of eye.

***Antennae*** moderately long, scapes slightly exceeding anterior margin of pronotum and longer than funicle, weakly regularly curved, in apical half slightly gradually thickened to apex, at apex 0.7–0.8 × as wide as club. Funicle segment 1 bead-shaped, 1.5–1.6 × longer than wide and 1.3–1.4 × longer than segment 2, which is 1.5–1.6 × longer than wide; segments 3–7 successively wider, segment 3 and 4 1.3–1.4 ×, segment 5–6 1.6–1.7 ×, segment 7 1.9–2.0 × wider than long. Clubs ovoid, large, 1.4–1.5 × longer than wide.

***Pronotum*** (Figs 19, 20) 1.17–1.22 × wider than long, widest at midlength, with distinctly rounded sides; anterior margin distinctly narrower than posterior one; disc flatly and irregularly granulate; in lateral view pronotum slightly convex, anterior margin strongly obliquely directed back beneath towards coxae.

***Elytra*** (Figs 19, 20) oval, 1.23–1.29 × longer than wide, widest at midlength, with regularly rounded sides. Striae coarsely punctate, wider than intervals, striae only slightly impressed between punctures; separation of punctures much shorter than their diameters. Intervals very narrow, somewhat convex, smooth.

***Protibiae*** (Fig. 40) short and robust, 4.8–5.2 × longer than wide at midlength, at apical quarter indistinctly curved inwards with mesal edge slightly bisinuate, apically bluntly truncate, with dense fringe of fine but long yellowish setae, shorter in mesal than in lateral part and with long and slender yellowish mucro. Tarsi short, tarsomere 2 1.4–1.5 × wider than long; tarsomere 3 1.2–1.3 × wider than long and 1.3–1.4 × wider than tarsomere 2; onychium (tarsomere 5) 1.1 × as long as tarsomere 3, widened apicad with very long, strongly divaricate claws, almost as long as exceeding part of onychium.

***Abdominal ventrites*** 1.09–1.12 × longer than wide, sparsely roughly punctate; ventrite 2 slightly longer than ventrite 1 and distinctly longer than ventrites 3 and 4 combined; suture between ventrites 1 and 2 sinuous, the others straight. Metaventral process as wide as transverse diameter of metacoxa.

***Penis*** (Fig. 42) short, 1.57 × as long as wide, subparallel-sided, slightly evenly enlarged apicad, in apical part shortly subtriangular, tip rounded, sides of tip shallowly concave; in lateral view moderately wide, ventral side almost straight, dorsal side irregularly rounded, tip pointed and curved upwards.

***Female genitalia.*** Spermatheca with short and moderately wide cornu; corpus large, elongated; ramus and collum developed, identically sized, short and wide (Fig. 44). Sternite VIII with plate 1.5–1.6 × longer than wide, rhombic, without any fenestra (Fig. 48). Gonocoxites of ovipositor very slender and long, basally enlarged, in apical part rod-shaped, bearing slender and long, cylindrical stylus with apical setae.

#### Bionomics.

The majority of the material was collected by sifting wet debris under trees along the border of an old orchard.

#### Etymology.

The new species is dedicated to one of the collectors and a very good friend of the second author, Dr. Jan Růžička (University of Life Science, Prague). The Czech name Jan has its nickname “Honza”. The specific name is a noun in apposition.

**Figures 26–35.**
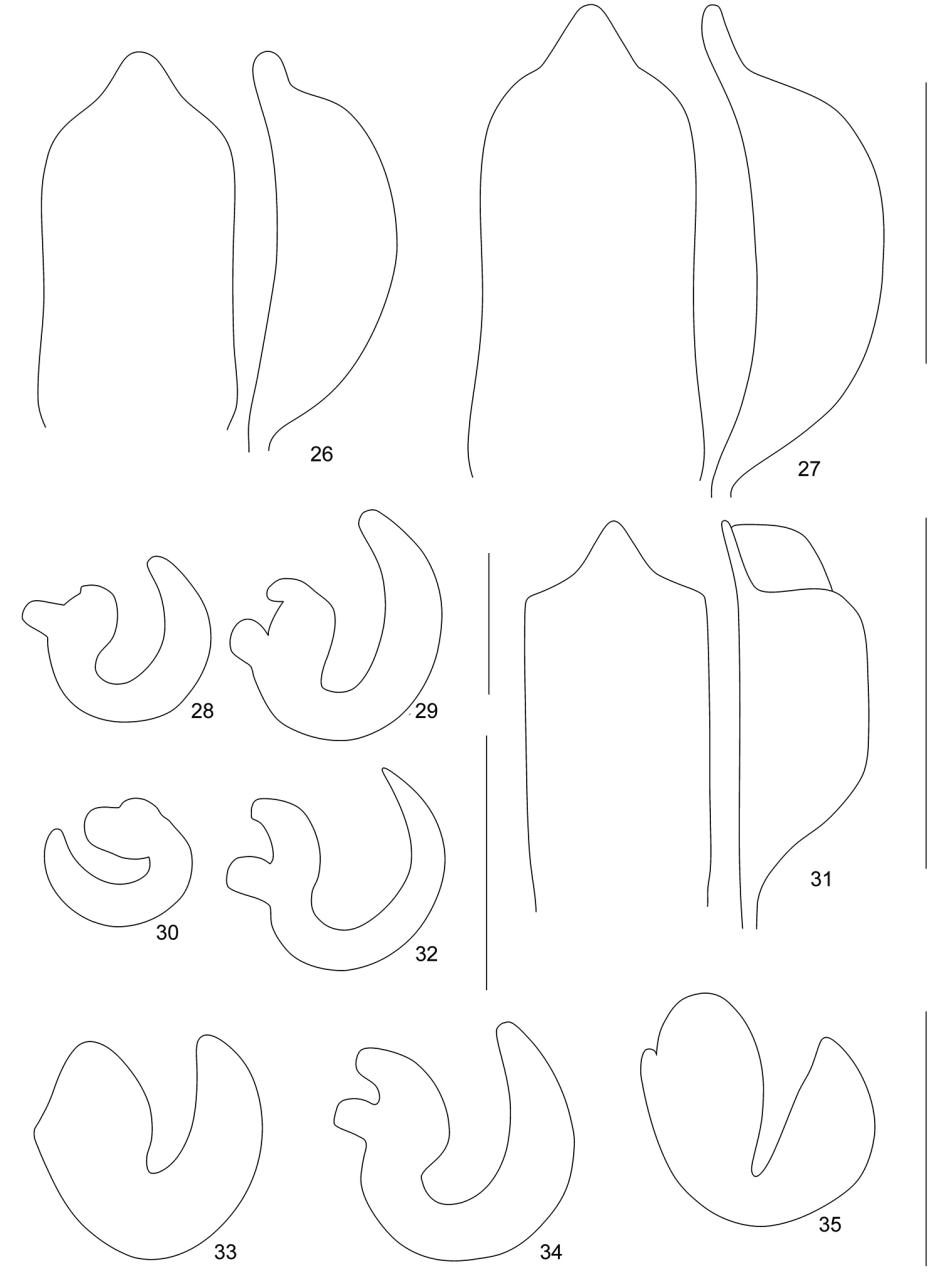
Genitalia of *Rhinodontodes* and *Rhinodontus*: **26** Penis of *Rhinodontodes alashanensis* sp. nov., dorsal and lateral view **27** Penis of *Rhinodontodes subsignatus*, dorsal and lateral view **28** Spermatheca of *Rhinodontodes alashanensis* sp. nov. **29** Spermatheca of *Rhinodontodes subsignatus***30** Spermatheca of *Rhinodontus crassiscapus***31** Penis of *Rhinodontus ignarus*, dorsal and lateral view **32** Spermatheca of *Rhinodontus ignarus***33** Spermatheca of *Rhinodontus mongolicus***34** Spermatheca of *Rhinodontus proximus***35** Spermatheca of *Rhinodontus sawadai*. Scale bars: 0.50 mm (**26, 27, 31**); 0.25 mm (**28–30, 32–35**).

#### Distribution.

China, Yunnan (Fig. 54).

#### Differential diagnosis.

*Trachyphloeosoma honza* sp. nov. shares with *T. martin* sp. nov. short and robust protibiae, short and wide rostrum and subspatulate setae. It is easily distinguished from *T. martin* sp. nov. by elytral setae on all elytral intervals, dorsal margin of antennal scrobes directed towards middle of eye and female sternite VIII lacking fenestra, while *T. martin* sp. nov. has elytral setae only on odd intervals, dorsal margin of scrobes directed above dorsal margin of eye and female sternite VIII with longitudinal fenestra. In comparison with non-Chinese species, *T. honza* sp. nov. is similar to *T. advena* Zimmerman, 1956, known from Japan, Korea and introduced to U.S.A. and *T. ryukyuensis* Morimoto, 2015, known from Japan, in the funicle being 7-segmented and body covered by appressed setae and elytra with raised setae on all intervals. It is possible to distinguish *T. honza* sp. nov. from both by short subspatulate setae, distinctly shorter than width of an elytral interval (long piliform setae on elytra, approximately as long as width of interval in *T. advena* and *T. ryukyuensis*), elytral setae distinctly bent backwards in lateral view (perpendicularly erect in *T. advena* and *T. ryukyuensis*) and plate of sternite VIII in females without fenestra (with fenestra in *T. advena* and *T. ryukyuensis*).

### Trachyphloeosoma jirka sp. nov.

Taxon classificationAnimaliaColeopteraCurculionidae

38BF82DD-1327-54EE-BFAE-7167A564531B

http://zoobank.org/766B872B-D8F0-4E20-8DD4-D27E44BAEA02

Figs 21 , 22 , 37 , 41 , 45 , 49 , 54

#### Type locality.

China, Jiangxi, Jinggangshan Mts., Xiangzhou.

#### Material examined.

***Holotype.*** China – **Jiangxi Prov.** • 1 ♀; Jinggangshan Mts., Xiangzhou (forested valley S of the village); 26°35.5'N, 114°16.0'E; 374 m a.s.l.; 26 Apr. 2011; Fikáček & Hájek leg.; sifting, accumulation of moist leaf litter along the stream and on the steep slope above the stream in the sparse secondary forest; [MF08]; NMPC. ***Paratypes.*** China – **Jiangxi Prov.** • 1 ♀; the same data as holotype; NMPC; • 1 ♀; same data as holotype; IZCAS.

#### Description.

Body length: 2.06–2.44 mm, holotype 2.06 mm.

***Body*** (Figs 21, 22) including antennae and legs unicoloured piceous brown. The entire body except for frons, antennal funicles with clubs and tarsi covered with a brownish earth-like incrustation which conceals most of the surface; appressed scales, covering the whole body, with hardly visible shape, but in lateral parts rounded, finely densely striolate. Elytra with one conspicuous, dense row of long erect setae on each interval, starting from the base; setae as long as width of one interval, very slender, slightly and evenly enlarged apicad, distance between two setae slightly longer than length of one seta. Pronotum and head with rostrum with identically long and shaped setae as elytral setae, densely irregularly scattered, anteriorly directed. Antennal scapes, femora and tibiae with long, erect, very slender setae, distinctly prominent from outline of scapes and legs.

***Rostrum*** (Figs 21, 22, 37) 1.12–1.18 × wider than long, at base 1.18–1.20 × wider than at apex, evenly tapered anteriad, at basal half with straight sides; in profile moderately long and slender, convex, at apex distinctly declined. Epifrons in basal half distinctly tapered anteriad, in apical half almost parallel-sided, narrow, 0.61–0.67 × as wide as rostrum in corresponding part, with ill-defined, slender, longitudinal furrow. Frons conspicuous, smooth, shiny, angularly declined from epifrons. Epistome small, short, indistinct, just at apical portion of rostrum, posteriorly narrowly carinate. Antennal scrobes in dorsal view visible as wide furrows, reaching eyes; in lateral view distinctly subtriangular, strikingly enlarged posteriad with dorsal margin directed above dorsal margin of eye and ventral margin deeply below ventral margin of eye. Eyes small, in dorsal view hardly protruding from outline of head; in lateral view placed subdorsally, distance from dorsal margin of head shorter than diameter of eye.

***Antennae*** moderately long, scapes slightly exceeding anterior margin of pronotum and distinctly longer than funicle, weakly curved in basal third, in apical half slightly gradually thickened to apex, at apex 0.7–0.8 × as wide as club. Funicle segment 1 bead-shaped, 1.3–1.4 × longer than wide and 1.4–1.5 × longer than segment 2, this is short, 1.1–1.2 × longer than wide; segments 3–7 slightly successively wider, segment 3 and 4 1.3–1.4 ×, segment 5–6 1.5–1.6 ×, segment 7 1.7–1.8 × wider than long. Clubs ovoid, large, 1.6–1.7 × longer than wide.

***Pronotum*** (Figs 21, 22) 1.21–1.28 × wider than long, widest at anterior third, with distinctly rounded sides, slightly constricted behind anterior margin; disc flatly and irregularly granulate, among granules irregularly punctate with rough and fine punctures; in lateral view pronotum slightly convex, anterior margin strongly obliquely directed back beneath towards coxae.

***Elytra*** (Figs 21, 22) oval, 1.42–1.46 × longer than wide, widest at midlength, with regularly rounded sides. Striae coarsely punctate, twice as wide as intervals, striae not impressed between the punctures; separations of punctures much less than their diameters. Intervals very narrow, flat, shiny.

***Protibiae*** (Fig. 41) long and slender, 6.1–6.3 × longer than wide at midlength, at apical quarter conspicuously curved inwards with mesal edge slightly bisinuate, apically obliquely subtruncate, with dense fringe of fine but long yellowish setae, shorter in mesal than in lateral part, with long and slender yellowish mucro. Tarsi short, tarsomere 2 1.4–1.5 × wider than long; tarsomere 3 1.3–1.4 × wider than long and 1.4–1.5 × wider than tarsomere 2; tarsomere 5 1.1 × as long as tarsomere 3, evenly widened apicad with very long, strongly divaricate claws, approximately as long as part of onychium (tarsomere 5) projecting beyond lobes of tarsomere 3.

***Abdominal ventrites*** sparsely roughly punctate; ventrite 2 slightly longer than ventrite 1 and distinctly longer than ventrites 3 and 4 combined; suture between ventrites 1 and 2 sinuate, the others straight. Metaventral process as wide as transverse diameter of metacoxa.

***Female genitalia.*** Spermatheca with very slender and irregularly distorted cornu; corpus large, elongate; ramus not developed; collum very small, hump-shaped, shorter than wide (Fig. 45). Sternite VIII with plate 2.0–2.2 × longer than wide, rhombic, without any fenestra (Fig. 49). Gonocoxites of ovipositor very slender and long, basally enlarged, in apical part rod-shaped, bearing slender and long cylindrical stylus with apical setae.

**Figures 36–41.**
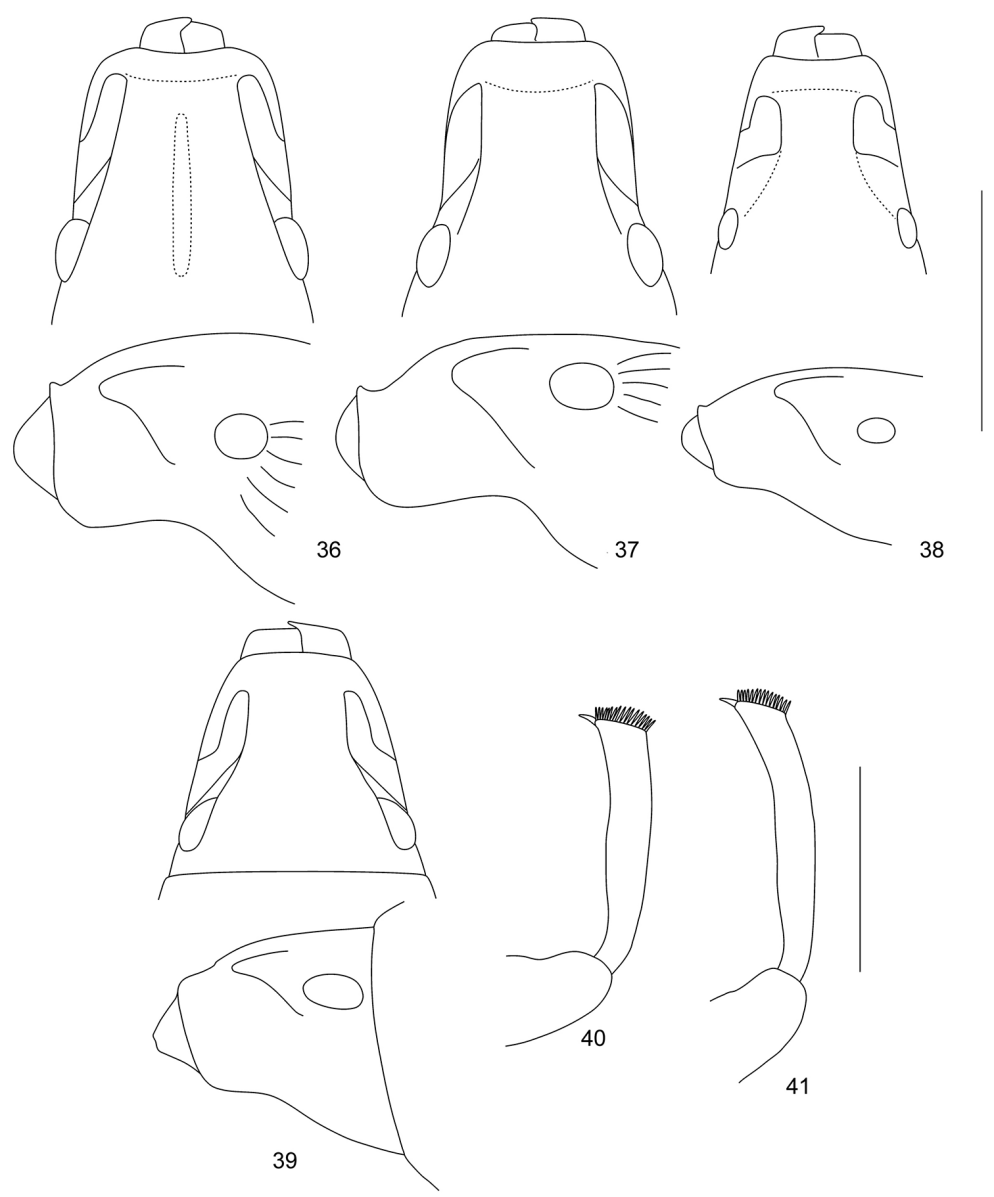
Head with rostrum in dorsal and lateral view of *Trachyphloeosoma* species: **36***T. honza* sp. nov. **37***T. jirka* sp. nov. **38***T. martin* sp. nov. **39***T. roelofsi*; Protibiae of *Trachyphloeosoma* species: **40***T. honza* sp. nov. **41***T. jirka* sp. nov. Scale bars: 0.50 mm (**36–41**).

#### Bionomics.

This species was collected by sifting in sparse secondary forest.

#### Etymology.

This species is dedicated to Dr. Jiří Hájek, curator of National Museum in Prague, who loaned us very interesting material of *Trachyphloeosoma* for study and also collected the specimens of this species. The nickname of Jiří is “Jirka” in the Czech language. The specific name is a noun in apposition.

#### Distribution.

China, Jiangxi (Fig. 54).

#### Differential diagnosis.

*Trachyphloeosoma jirka* sp. nov. is easily distinguishable among Chinese *Trachyphloeosoma* species by its long and slender protibiae, distinctly curved inwards at apical part, long piliform setae as long on pronotum as on elytra, long and slender rostrum with frons distinctly declined downwards, subdorsal eyes and long and slender plate of female sternite VIII. In comparison with non-Chinese species, *T. jirka* sp. nov. is, in the funicle 7-segmented, body covered by appressed setae and elytra with raised setae on all intervals similar to *T. advena* Zimmerman, 1956, known from Japan, Korea and introduced to U.S.A. and *T. ryukyuensis* Morimoto, 2015, known from Japan. It is possible to distinguish it from both by erect setae on pronotum equal in length to elytral setae (distinctly shorter in *T. advena* and *T. ryukyuensis*), elytra long, oval, 1.42–1.46 × longer than wide (oval, 1.26–1.31 × longer than wide long in *T. advena* and *T. ryukyuensis*) and protibiae slender, distinctly curved inwards at apical portion (short and robust, only slightly curved in *T. advena* and *T. ryukyuensis*) and also plate of sternite VIII in females without fenestra (with fenestra in *T. advena* and *T. ryukyuensis*).

### Trachyphloeosoma martin sp. nov.

Taxon classificationAnimaliaColeopteraCurculionidae

8CDC37CF-558E-5FBC-A722-D174303EC669

http://zoobank.org/0F81305F-227E-49C0-95E3-B4FB14BB3F1C

Figs 23 , 24 , 38 , 43 , 46 , 50 , 54

#### Type locality.

China, Hainan, Limushan Mts.

#### Material examined.

***Holotype.*** China – **Hainan Prov.** • 1 ♂; Limushan Mts., mountains above frst. admin. Centre; 19°10.5–19°10.9'N, 109°44–109°45'E; 650–900 m a.s.l.; 6 May 2011; Fikáček leg.; sifting – small accumulations of moist leaf litter along an on the trail in secondary forest partly with *Cyathea* and bamboo; MF19; NMPC. ***Paratypes.*** China – **Hainan Prov.** • 1 ♀; the same data as holotype; NMPC; • 1 ♀; same data as for preceding; IZCAS.

#### Description.

Body length: 1.63–2.31 mm, holotype 1.63 mm.

***Body*** (Figs 23, 24) including antennae and legs unicoloured piceous brown. Entire body except of frons, antennal funicles with clubs and tarsi covered with a brownish earth-like incrustation which conceals integument; rounded scales with hardly visible shape, but at least on pronotum, head and rostrum irregularly star-shaped. Elytra with one conspicuous dense row of erect, subspatulate setae only on odd-numbered intervals; setae almost as long as width of one interval, enlarged apicad, distance between two setae distinctly longer than length of one seta. Pronotum and head with rostrum with similar setae, approximately half length of elytral setae, densely irregularly scattered, anteriorly directed. Antennal scapes, femora and tibiae with short, erect, very slender setae, prominent from outline of scapes and legs.

***Rostrum*** (Figs 23, 24, 38) 1.38–1.42 × wider than long, at base 1.23–1.28 × wider than at apex, evenly tapered anteriad with straight sides; in profile short and wide, convex. Epifrons in basal half distinctly tapered anteriad, in apical half almost parallel-sided, narrow, 0.62–0.67 × as wide as rostrum in corresponding part, with ill-defined, longitudinal furrow. Frons glabrous, smooth and shiny, posteriorly continuous with epifrons. Epistome indistinct. Antennal scrobes in dorsal view visible as furrows, not reaching eyes; in lateral view distinctly subtriangular, short, strikingly enlarged posteriad with dorsal margin directed above dorsal margin of eye and ventral margin deeply below ventral margin of eye. Eyes very small, in dorsal view hardly protruding from outline of head; in lateral view placed in dorsal third, distance from dorsal margin of head distinctly longer than diameter of eye.

***Antennae*** moderately long, scapes slightly exceeding anterior margin of pronotum and longer than funicle, weakly regularly curved, in apical half slightly gradually thickened to apex, at apex 0.7–0.8 × as wide as club. Funicle segment 1 wide, bead-shaped, 1.3–1.4 × longer than wide and 1.6–1.7 × longer than segment 2, which is short, 1.1–1.2 × longer than wide; segments 3–7 slightly successively wider, segments 3–5 1.6–1.7 ×, segment 6 1.6–1.7 ×, segment 7 1.7–1.8 × wider than long. Clubs ovoid, large, 1.5–1.6 × longer than wide.

***Pronotum*** (Figs 23, 24) narrow, 1.07–1.11 × wider than long, widest at anterior third, with distinctly rounded sides, constricted behind anterior margin; disc regularly domed, indistinctly granulate; pronotum slightly convex in lateral view, anterior margin strongly obliquely directed back beneath towards coxae.

***Elytra*** (Figs 23, 24) elongated, 1.44–1.48 × longer than wide, widest at midlength, with regularly rounded sides. Striae coarsely punctate, twice as wide as intervals, striae not impressed between punctures; separation of punctures much less than their diameters. Intervals very narrow, flat, shiny.

***Protibiae*** short and robust, 5.0–5.3 × longer than wide at midlength, at apical quarter slightly curved inwards with mesal edge slightly bisinuate, apically obliquely subtruncate, with a dense fringe fine of long yellowish setae, shorter in mesal than in lateral part, with long and slender yellowish mucro. Tarsi short, tarsomere 2 1.6–1.7 × wider than long; tarsomere 3 1.3–1.4 × wider than long and 1.4 × wider than tarsomere 2; onychium (tarsomere 5) as long as tarsomere 3, strikingly widened apicad with very long, strongly divaricate claws, as long as part of onychium projecting beyond lobes of tarsomere 3.

***Abdominal ventrites*** 1.14–1.19 × longer than wide, sparsely roughly punctate; ventrite 2 slightly longer than ventrite 1 and distinctly longer than ventrites 3 and 4 combined; suture between ventrites 1 and 2 sinuate, the others straight. Metaventral process as wide as transverse diameter of metacoxa.

***Penis*** (Fig. 43) long and slender, 2.91 × as long as wide, subparallel-sided with straight sides, slightly evenly tapered apicad; tip long, subtriangular with slightly concave sides; in lateral view slender, distinctly irregularly curved, tip pointed.

***Female genitalia.*** Spermatheca with long and irregularly curved cornu; corpus slender, indistinct; ramus developed, short or tubular; collum very long, distinctly irregularly curved (Fig. 46). Sternite VIII with plate 1.5–1.7 × longer than wide, rhombic, with distinct, slender, longitudinal fenestra reaching midlength of plate (Fig. 50). Gonocoxites of ovipositor very slender and long, basally enlarged, in apical part rod-shaped, bearing slender and long, cylindrical stylus with apical setae.

**Figures 42–51.**
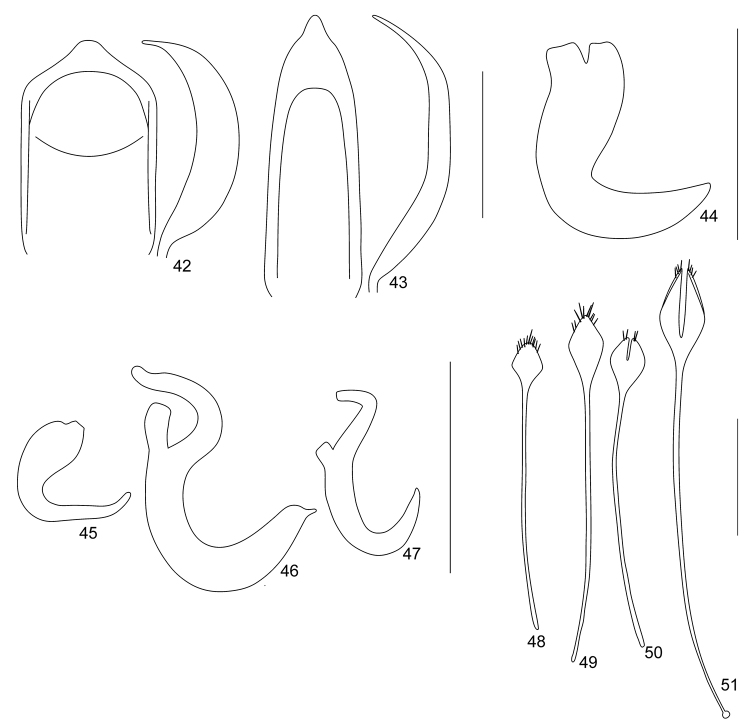
Terminalia of *Trachyphloeosoma* species: **42** Penis of *T. honza* sp. nov., dorsal and lateral view **43** Penis of *T. martin* sp. nov., dorsal and lateral view **44** Spermatheca of *T. honza* sp. nov. **45** Spermatheca of *T. jirka* sp. nov. **46** Spermatheca of *T. martin* sp. nov. **47** Spermatheca of *T. roelofsi***48** Sternite VIII of *T. honza* sp. nov. **49** Sternite VIII of *T. jirka* sp. nov. **50** Sternite VIII of *T. martin* sp. nov. **51** Sternite VIII of *T. roelofsi*. Scale bars: 0.25 mm (**42–47**); 0.5 mm (**48–51**).

#### Bionomics.

Type material was sifted from leaf litter in secondary forest partly with *Cyathea* and bamboo.

#### Etymology.

This species is named after the curator of the National Museum in Prague and also the collector of the type specimens, Dr. Martin Fikáček. The specific name is a noun in apposition.

#### Distribution.

China, Hainan (Fig. 54).

#### Differential diagnosis.

*Trachyphloeosoma martin* sp. nov. is very easily recognizable among Chinese species by the elytral raised setae only on odd-numbered intervals and also by the pronotum being somewhat longer, only slightly wider than long, not distinctly granulate on disc, almost flat. Within the genus, *Trachyphloeosoma martin* sp. nov. is similar only to *T. roelofsi* Sharp, 1896 from Japan and *T. setosum* (Wollaston, 1869) known from St. Helena, where it is apparently introduced (but region of origin not yet known). *Trachyphloeosoma martin* sp. nov. is similar to them in having raised elytral setae only on odd intervals, but distinguished from them by a more slender and longer rostrum, 1.38–1.42 × wider than long (1.56–1.73 × in *T. roelofsi* and *T. setosum*), longer and more slender elytra, 1.44–1.48 × longer than wide, (1.19–1.27 × in *T. roelofsi* and *T. setosum*), and also by the different shape of the spermatheca, with collum distinctly longer than wide (isodiametric in *T. roelofsi*), or long and irregularly curved cornu (short and regularly curved in *T. setosum*).

### Trachyphloeosoma roelofsi

Taxon classificationAnimaliaColeopteraCurculionidae

Sharp, 1896

6CE3DF4D-8704-5EC3-BD3F-D218E9B9E146

Figs 39 , 47 , 51

Trachyphloeops setosus Roelofs 1873: 166 (**non** Wollaston, 1869).
Trachyphloeosoma roelofsi Sharp, 1896: 92 (nomen novum for Trachyphloeops setosus Roelofs); Morimoto 2015: 346 (review of Japanese species).
Trachyphloeosoma setosum : Zimmerman 1956: 27 (review of genus); Borovec 2009: 78 (check-list); Borovec 2014: 20 (revision of genus); Alonso-Zarazaga et al. 2017: 403 (catalogue).

#### Material examined.

China – **Taiwan** • 1 ♀; TianMu Gudao Hik. Trail (Taipei) Beitou Twnsh., Taipei Co., S. Samau Mt.; 3 Jan. 2009; S. Vít leg.; dead leaves; NMPC; • 1 ♀; Rd. Jhuzihhu/Shuiwei, Yangmingshan Mts., slopes E of Mt. Datun, Taipei Co.; 650 m a.s.l.; 24 Oct. 2007; S. Vít leg.; putresc. base of Cryptomeria (?); NMPC.

#### Remarks.

This species was described by Sharp from Nagasaki, Japan. Zimmerman (1956) compared the series of *Trachyphloeosoma setosum* Wollaston, 1869 from St. Helena with Sharp´s material of *T. roelofsi* from Japan and found the two series represent only one species and placed them in synonymy. However, Morimoto (2015) resurrected the name *T. roelofsi* as an independent species, and distinguished it from *T. setosus* Wollaston from St. Helena. *Trachyphloeosoma roelofsi* is thus known from Japan and Taiwan, while *T. setosus* is assumed as a species introduced to St. Helena without knowledge of its original country.

**Figure 52.**
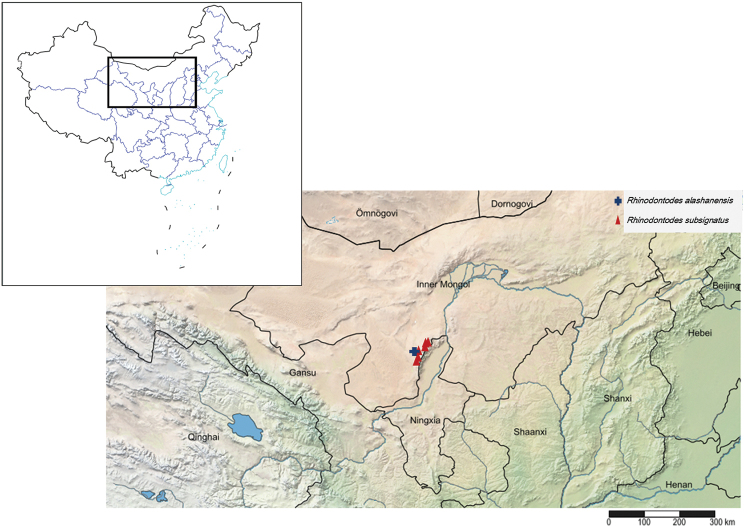
Geographical distribution of new species and new record of *Rhinodontodes* in China.

**Figure 53.**
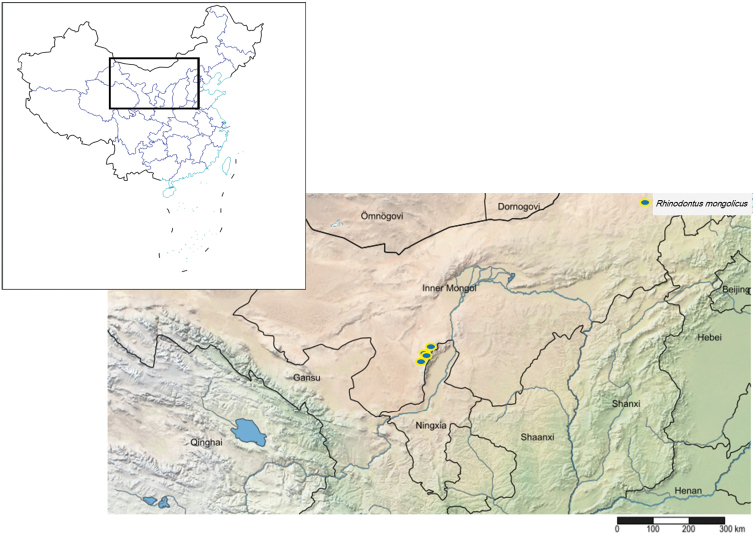
Geographical distribution of new record species of *Rhinodontus* in China.

**Figure 54.**
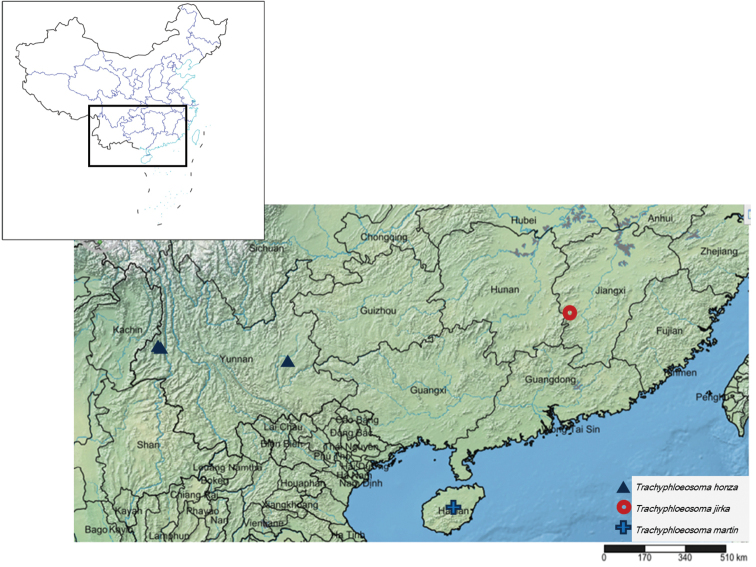
Geographical distribution of new species of *Trachyphloeosoma* in China.

### Key to Chinese *Trachyphloeosoma* species

**Table:** 

1	Protibiae long and slender, 6.1–6.3 × longer than wide at midlength, at apical portion distinctly inwardly curved (Fig. 41). Elytral setae piliform, as long as setae on pronotum (Fig. 22). Rostrum long and slender, 1.12–1.18 × wider than long (Fig. 37). Frons distinctly declined from epifrons (Fig. 37). Distance between head and eye shorter than diameter of eye in profile (Fig. 37). Plate of female sternite VIII more elongate, rhombic, 2.0–2.2 × longer than wide, lacking fenestra (Fig. 49). China: Jiangxi	***T. jirka* sp. nov.**
–	Protibiae short and robust, 4.8–5.3 × longer than wide at midlength, at apical portion only slightly inwardly curved (Fig. 40). Elytral setae subspatulate, twice as long as setae on pronotum (Figs 20, 24). Rostrum short and wide, 1.25–1.42 × wider than long (Figs 36, 38, 39). Frons continuous with epifrons (Figs 36, 38, 39). Distance between head and eye longer than diameter of eye in profile (Figs 36, 38, 39). Plate of female sternite VIII proportionally shorter, rhombic, 1.5–1.7 × longer than wide, if twice longer than wide, then with fenestra (Figs 48, 50, 51)	**2**
2	Elytral setae on all elytral intervals, half as long as width of interval (Fig. 19). Epifrons with straight sides (Fig. 36). Dorsal margin of antennal scrobes directed towards middle of eye (Fig. 36). Pronotum shorter, 1.17–1.22 × wider than long, widest at midlength, distinctly granulate (Fig. 36). Penis short and wide, in profile wide (Fig. 42). Spermatheca with ramus and collum equally sized (Fig. 44). Female sternite VIII lacking fenestra (Fig. 48). China: Yunnan	***T. honza* sp. nov.**
–	Elytral setae on odd-numbered elytral intervals only, almost as long as width of interval (Fig. 23). Epifrons with concave sides (Figs 38, 39). Dorsal margin of antennal scrobes directed above eye (Figs 38, 39). Pronotum slightly longer, 1.07–1.15 × wider than long, widest at anterior third, almost flat (Fig. 23). Penis long and slender, slender in profile (Fig. 43). Spermatheca with collum distinctly longer than ramus (Figs 46, 47). Female sternite VIII with distinct slender fenestra (Figs 50, 51)	**3**
3	Rostrum more slender, 1.38–1.42 × wider than long (Fig. 38). Eyes smaller, in profile with greater distance from dorsal margin of head (Fig. 38). Elytra oval, 1.44–1.48 × longer than wide. Spermatheca with ramus distinctly longer than wide (Fig. 46). Female sternite VIII with plate 1.5–1.7 × longer than wide and fenestra reaching midlength of plate (Fig. 50). China: Hainan	***T. martin* sp. nov.**
–	Rostrum wider, 1.56–1.73 × wider than long (Fig. 39). Eyes larger, in profile nearer to dorsal margin of head (Fig. 39). Elytra suboval, 1.19–1.27 × longer than wide. Spermatheca with ramus isodiametric (Fig. 47). Female sternite VIII with plate twice as long as wide and fenestra reaching basal part of plate (Fig. 51). China, Taiwan; Japan	***T. roelofsi* Sharp**

### Key to Chinese genera of Trachyphloeini

**Table:** 

1	Anterior margin of pronotum with postocular lobes in lateral view. Claws connate in short basal part	**2**
–	Anterior margin of pronotum straight or oblique in lateral view, without postocular lobes. Claws free	**4**
2	Epistome conspicuous in dorsal as well as in lateral view, projecting as two teeth from outline of head. Postocular lobes with short fringe of setae	**3**
–	Epistome inconspicuous, not projecting from outline of the head. Postocular lobes without setae. 2.6–6.8 mm	***Pseudocneorhinus* Roelofs**
3	Rostrum before eyes enlarged, wider than long. Apex of protibiae enlarged laterally, with stout spines. Claws divaricate. Length 2.9–4.1 mm	***Rhinodontus* Faust**
–	Rostrum very feebly enlarged anteriad, almost parallel-sided, longer than wide. Apex of protibiae feebly enlarged laterally, with slender, bristle-shaped spines. Claws almost parallel-sided, feebly divaricate. Length 3.8–4.9 mm	***Rhinodontodes* Voss**
4	Genae glabrous, longitudinally striate. Antennal scrobes in lateral view gently enlarged posteriad, dorsal margin directed towards ventral margin of eye. Rostrum as long as wide, or slightly wider than long. Length 3.0 mm	***Trachyphilus* Faust**
–	Genae squamose, lacking striae. Antennal scrobes in lateral view enlarged posteriad, subtriangular; dorsal margin directed towards dorsal margin of eye. Rostrum wider than long. Length 1.7–2.3 mm	***Trachyphloeosoma* Wollaston**

## Supplementary Material

XML Treatment for Rhinodontodes

XML Treatment for Rhinodontodes alashanensis

XML Treatment for Rhinodontodes subsignatus

XML Treatment for Rhinodontus

XML Treatment for Rhinodontus crassiscapus

XML Treatment for Rhinodontus ignarus

XML Treatment for Rhinodontus mongolicus

XML Treatment for Rhinodontus proximus

XML Treatment for Rhinodontus sawadai

XML Treatment for Trachyphloeosoma

XML Treatment for Trachyphloeosoma honza

XML Treatment for Trachyphloeosoma jirka

XML Treatment for Trachyphloeosoma martin

XML Treatment for Trachyphloeosoma roelofsi
